# Implications of Intravital Imaging of Murine Germinal Centers on the Control of B Cell Selection and Division

**DOI:** 10.3389/fimmu.2016.00593

**Published:** 2016-12-19

**Authors:** Sebastian C. Binder, Michael Meyer-Hermann

**Affiliations:** ^1^Department of Systems Immunology, Braunschweig Integrated Centre of Systems Biology, Helmholtz Centre for Infection Research, Braunschweig, Germany; ^2^Institute for Biochemistry, Biotechnology and Bioinformatics, Technische Universität Braunschweig, Braunschweig, Germany

**Keywords:** germinal center, antibody optimization, affinity maturation, B cell motility, T follicular helper cells, chemotaxis, dark and light zone

## Abstract

Intravital imaging of antibody optimization in germinal center (GC) reactions has set a new dimension in the understanding of the humoral immune response during the last decade. The inclusion of spatio-temporal cellular dynamics in the research on GCs required analysis using the agent-based mathematical models. In this study, we integrate the available intravital imaging data from various research groups and incorporate these into a quantitative mathematical model of GC reactions and antibody affinity maturation. Interestingly, the integration of data concerning the spatial organization of GCs and B cell motility allows to draw conclusions on the strength of the selection pressure and the control of B cell division by T follicular helper cells.

## 1 Introduction

Germinal centers (GCs) are specialized environments normally located in lymphoid tissues giving rise to high-affinity antibodies (1, 2). This process depends on antigen presented on Fc receptors on follicular dendritic cells (FDCs) and involves mutation of the antigen-binding region of B cell receptors (3), competitive collection of antigen (4, 5), and competition for help from T cells (6–8). As it is rather unlikely to find an optimal antibody with about 5–10 mutations from the germline sequence in a single shot, GCs select B cells in cycles of mutation and selection, as was predicted from mathematical modeling (9). This recycling process of re-proliferation after successful selection was further predicted to be the dominant fate of B cells: more than 80% of the selected B cells return to the state of mutation and have to be selected again (10). While this exciting invention of nature was derived from theoretical investigations, it already entered the text book of GC in 1994 (1).

GCs exhibit a specific spatial organization in dark zone (DZ) and light zone (LZ) (11), which is due to highly motile B and T cells, respectively. While T cells are mostly found in the LZ (12), B cells are sorted according to the differentiation state: dividing and mutating B cells are concentrated in the DZ while B cells in the state of competitive selection are in the LZ. Thus, the recycling of B cells described above is associated with migration between these two zones.

In GCs, the LZ is dominated by the chemokine CXCL13 generated by FDCs, while the DZ is mostly CXCL12-positive, which stems from stromal cells located at the boundary of the DZ toward the T zone (13). The receptors of both chemokines, CXCR5 and CXCR4, are specifically expressed by the two subsets of B cells populating the LZ and the DZ, respectively, suggesting that B cells upon switching between the states of selection and division would regulate their expressed chemokine receptor and migrate to the opposite zone by chemotaxis. Indeed, CXCR4 expression is used as a common marker to identify GC-DZ B cells (14).

The motility of B and T cells in lymphoid tissue was first measured with two-photon imaging in the pioneering work of Mark Miller, who found a linear relationship between the distance reached and square root of time in both cell types (15), suggesting movement by random walk. Subsequently, this methodology was directly applied to GC B cells by three groups of investigators (6, 16, 17), who, surprisingly, found random walk again, thus, speaking against GC B cells following a chemokine gradient. The measurements of distance reached were found to be not sufficiently sensitive to small contributions of chemotaxis. The transient chemotaxis model emerged from these data (18), which postulates that B cells are desensitized within the zones for their respective chemokine and are only driven back to the target zone when random walk would drive them out of the zone.

The same three groups of investigators further investigated whether B cells would really permute between both zones as postulated by the recycling hypothesis. They found a comparably low frequency of transzone migration events of 5% with less migration events from the LZ to the DZ (6, 16, 17). The time window for cell tracking of 1 h or less was short when compared to the time scale of the processes occurring in the GC reaction (19). It was shown by mathematical modeling that these low frequencies and the directional asymmetry are compatible with the recycling model (18). The most convincing proof of recycling was provided by photoactivation of GC B cells in either zone or following their tracks for 6 h (8), which is the right duration of experiments for the analysis of transzone migration. Furthermore, it was shown that B cells artificially overloaded with antigen were leading to a burst of division in the DZ, suggesting that previously selected B cells really return to the DZ for further division (8).

The GC B cell growth in the DZ after incorporation of high amounts of antigen in the LZ (8) led to the prediction that the number of divisions induced in positively selected B cells after interaction with T follicular helper (Tfh) cells is regulated depending on the amount of pMHC presented on the B cell (20), which reflects the affinity of the B cell receptor for the antigen. We denoted this mechanism as a dynamic number of divisions (DND). The DND was confirmed in the experiment done by Gitlin et al. (21).

Both the spatio-temporal dynamics of GCs and two-photon imaging of cell motility are perfect topics for mathematical modeling. With the help of agent-based models, the motility of the constituents of the GC reaction can be analyzed and several predictions generated in this way were confirmed. The fact that the GC is a comparably closed system attracted many physicists, because it avoided the problems with open systems, which are more difficult to predict but common to biological systems. However, in recent years, the GC was revealed to be less closed than thought before: not only naive B cells migrate between follicles (17) but also Tfh cells migrate between different GCs (22). It was found that the number of clones mounting a GC reaction is not in the range of 3–5 (23, 24) but, depending on the antigen used for immunization, would reach up to 200 founder clones (25), which suggests that new founder clones would enter already mounted GC reactions. Furthermore, soluble high-affinity antibodies that are derived from the GC product, namely the plasma cells, feed back onto the GC reaction itself by covering the antigen presented on the FDC network (26).

These recent developments led to an extension of the mathematical models to include antibody feedback, DND (27), and continuous influx of founder clones into GCs (25). In the first step, we provide some technical details of the formulation of the GC simulation and describe limitations in the interpretation from the chosen lattice-based approach. Then, in view of the tight connection between the spatial dynamics of GC B cells and B cell selection, we asked whether it would be possible to derive the strength of competition and the number of induced B cell divisions after recycling from the integrated two-photon data on B cell motility in GCs.

The significant changes in the model represent the inclusion of a large body of new research. However, it has not been investigated whether this updated model is still in agreement with current experimental data. Hence, we investigate here whether the updated model can reproduce data from different sources. Furthermore, DND has been confirmed by experimental data but not yet quantified. Although previous versions of the model already include DND as a mechanism, no attempt was made to quantitatively characterize this mechanism previously. In this study, we show that DND is required to reproduce experimental results and specify the related model parameters for the first time.

## 2 Materials and Methods

For the simulation of cell motility and interactions in GC reactions, we use an agent-based modeling approach. The advantage in comparison to partial differential equations is the intuitive and accessible representation of objects rather than densities. This also allows for robust implementations based on object-oriented programming in C++. The basic structure of the LEDA model was described earlier (20) and hence here we only refer to the parts that have been added since then or which are particularly important for the description of two-photon motility data.

### 2.1 Space Discretization

While detailed analysis of motility data might benefit from a continuous space representation, we have chosen to use a lattice-based approach in order to achieve fast and feasible simulations of 21 days real-time GC reactions. We use a squared lattice in three dimensions with a lattice constant of 5 µm. Note that the resolution of the lattice impacts onto the analysis of motility measurements *in silico* (see Section 3).

The lattice is also used for the diffusion of chemokines. As the producers of the chemokine are fixed in space, we neglect consumption of chemokines by internalization of receptor–ligand complexes (28). The chemokine configuration was calculated from the distribution of FDCs in the FDC network and then saved for subsequent simulations. Again, this reduces the overall CPU load without harming the simulation results. Results are double checked with the full reaction–diffusion system at work.

### 2.2 Overview on GC Dynamics *In Silico*

The LEDA model is used (20), according to which founder B cells divide six times before first differentiation to the LZ phenotype. At each division, the B cells mutate with a probability of 0.3 (29). The LZ B cell searches for antigen on FDCs and binds it with a probability corresponding to the affinity of the B cell receptor for the antigen. The affinity is calculated from the position of the B cell in the discrete shape space relative to the position of the best possible antibody for this antigen (30). The LZ B cell stores the collected antigen. If no antigen is bound in a critical period, the cell is assumed to die by apoptosis. Successful B cells search for Tfh cells, which polarize to the B cell with the highest amount of collected antigen (assumed to reflect a higher amount of presented peptide MHC). The B cells integrate signals from the Tfh cell that is polarized toward it. If the B cell fails to collect sufficient signals within a critical time, it will again die by apoptosis. Thus, each LZ B cell has to survive two critical selection points: antigen collection and help from Tfh cells. Once a B cell survives both selection steps, it re-differentiates to the DZ phenotype and continues dividing and mutating, thereby they divide the collected antigen asymmetrically onto the daughter cells (31). At the end of the division phase, the DZ B cell with highest amount of antigen terminally differentiates to a plasma cell and leaves the GC area through the DZ in direction of the T–B zone boundary. Note that the amount of plasma cells generated in this model is 10-fold higher than that in traditional models where the B cells leave the GC after selection by Tfh (32).

### 2.3 Antibody Feedback

The antibodies produced by plasma cells that left the GC have an affinity to the antigen known *in silico*. Assuming that these antibodies are soluble and distribute over the whole organism and further assuming that all GCs in the organism generate antibodies in similar kinetics, one may multiply the number of generated plasma cells from the simulated GC by the total number of GCs in the organism. With a typical production rate per cell, the total amount of produced antibodies per time can be calculated. The antibodies are diluted over the whole organism and thus the concentration coming back to the simulated GC can be estimated.

Starting from this reflection, we modeled the amount of free antigen on the FDC network with chemical kinetics derived from the estimated concentration of antibodies over time. Only the free fraction of antigen is available to the B cells for collection (26).

### 2.4 Continuous Influx of B Cell Founder Cells

It was found that the number of B cells mounting a GC reaction is much larger than thought before (25), setting in question the previous model of oligoclonal GCs (23, 24). This finding raised the question of how long new founder cells would be incorporated into a starting GC. Based on the finding that there is a time window of a few days during which new GC reactions can be mounted (33), we estimated that the influx of new B cell clones into a GC would be limited to 4 days. The present simulations were done with an influx rate of 2 founder cells per hour, leading to 100–200 founder cells in each GC simulation.

### 2.5 Dynamic Number of Divisions

The prediction that the number of divisions induced in selected B cells would depend on the B cell efficiency in collecting and processing antigen (20) was confirmed by measurement of the effect of titrating the amount of antigen taken up by the B cells (21). This dependency is modeled with a Hill function describing how the number of divisions increases with increasing amount of collected antigen (27). However, the parameter values of this relationship were not identified so far and are subjected to this paper.

## 3 Results

Based on the new mathematical model of GC reactions including all the new features described above, we asked whether this simulation would still reproduce all of the results from two-photon measurements in GCs.

### 3.1 Reached Distance

Reached distance curves plot the distance of an object from its starting point over the square root of time. This curve is linear for random walk and quadratic for ballistic motion, which would correspond to chemotaxis in the present context. A persistent random walk starts with a quadratic curve and merges into a linear curve. The steepness of the linear part reflects the motility coefficient. Persistent random walk with a persistence time between 1 and 2 min was found in all two-photon experiments (6, 15–17).

**Figure 1:**
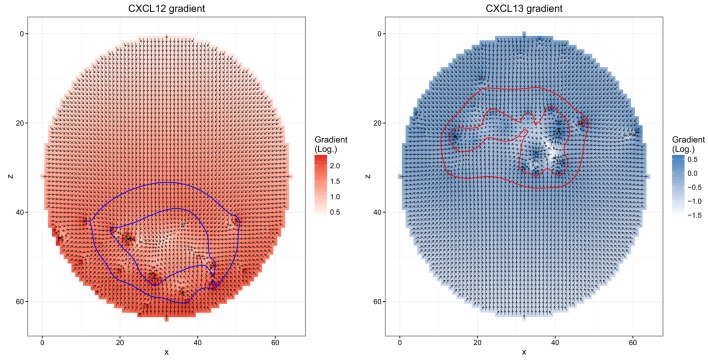
**Chemokine distributions and de-/resensitization**. Figures show the chemokine gradient in a two-dimensional cutsection through a germinal center. Gradient directions are indicated by the arrows in the vector field plots, its magnitude is shown by the color. Lines indicate the positions at which chemotaxis desensitization (inner border) and resensitization (outer border) occur.

The simulation uses mean speed and the measured persistence time as input. Chemokinesis is ignored. Chemotaxis is derived from soluble chemokine configurations. The persistence time defines the interval at which the cell would measure the chemokine configuration and determine a new direction of movement. The sensitivity of the cell for the chemokines is a dynamic quantity itself (Figure 1): when it encounters a critical chemokine concentration *c*_D_, it desensitizes and does random walk. It needs to find another critical chemokine concentration *c*_R_ < *c*_D_ to resensitize. When the cell is sensitive for the chemokine, the direction of movement is the random walk direction combined with the direction of the chemokine gradient [see Ref. (18) for more details of the transient chemotaxis model].

**Figure 2:**
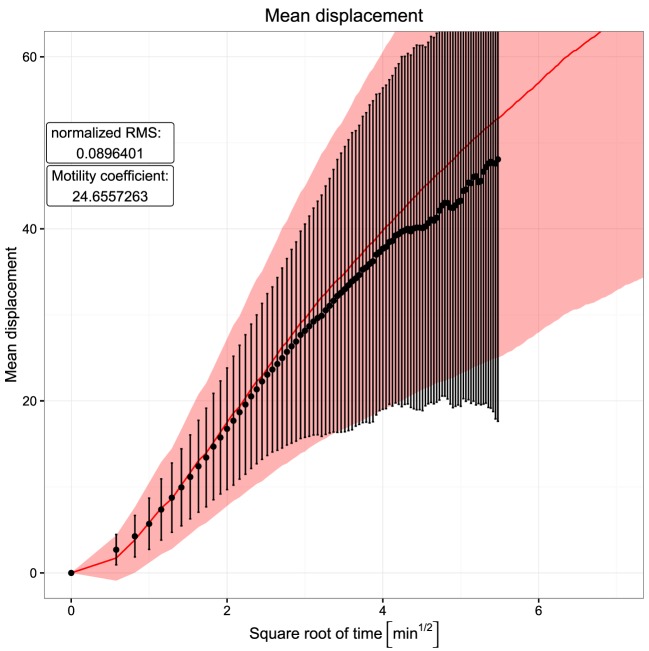
**Mean displacement versus square root of time**. Dots and error bars indicate the mean displacement and SD of tracked cells as measured in experiment (6). The red line and shaded area show mean displacement and SD of tracked cells in 20 simulations. The cell position is tracked every 20 s both *in silico* and in experiment.

With this simple model, persistent random walk is still found *in silico* despite the activity of chemotaxis (Figure 2). Using the measured mean, speed *in silico* leads to a reached distance curve and a motility coefficient consistent with the experimental results. The motility coefficient in a three-dimensional system can be calculated as:

(1) $$M=\frac{<|r_{i}(t)|{>}^{2}}{6t},$$

where <∣*r_i_*(*t*)∣> represents the mean displacement. *t* is the time interval in which the motility coefficient is measured. In order to get a realistic picture, it is important to limit this calculation to the linear part of the mean displacement curve, since the initial quadratic part is a result of the persistence time. Hence, the motility coefficient was calculated here from the slope of a linear regression line through the points in the time interval between 4 and 9 min. Its value of approximately 24.7 in the simulations is in the same range as the experimentally observed value of 21.4 reported in Ref. (6).

Note that the usage of a lattice with a lattice constant of 5 µm tends to hide the persistence time, i.e., the ballistic curve at short times in the reached distance plot. This is because the assumed mean speed of 7.5 µm/min induces a displacement or not of the cell by one full lattice constant. Surprisingly, the ballistic curve is still found *in silico* (Figure 2). By measurement of many cells *in silico* every 20 s (with same interval as in experiment), the initial quadratic part of the reached distance curve is observed by measuring not the time average of individual cells but the ensemble average of big steps on the lattice performed by many cells.

The choice of the lattice constant itself also has an impact onto the reached distance and motility. Increasing the lattice constant to 10 µm not only makes the steps (straight paths) per movement event larger but also induces a crowded microenvironment of the cells. This inhibits the overall motility and leads to a flat reached distance curve and a lower motility coefficient starting from the same *in silico* target speed. Thus, the choice of the lattice constant in the simulations is important to match the right migration behavior (see Section 3.4).

### 3.2 Speed Distribution

The cells tracked in two-photon experiments can be analyzed for the frequency of particular speeds. The positions of cells are measured at regular time intervals [for example, 20 s in (6)]. The resulting speed distribution strongly depends on the chosen time interval (18). Thus, the information that can be drawn from such distributions has to be interpreted in terms of the chosen time interval. The speed distribution cannot be interpreted as a property of the cells themselves but has to be considered as a read out that depends on a technical parameter of the measurement.

**Figure 3:**
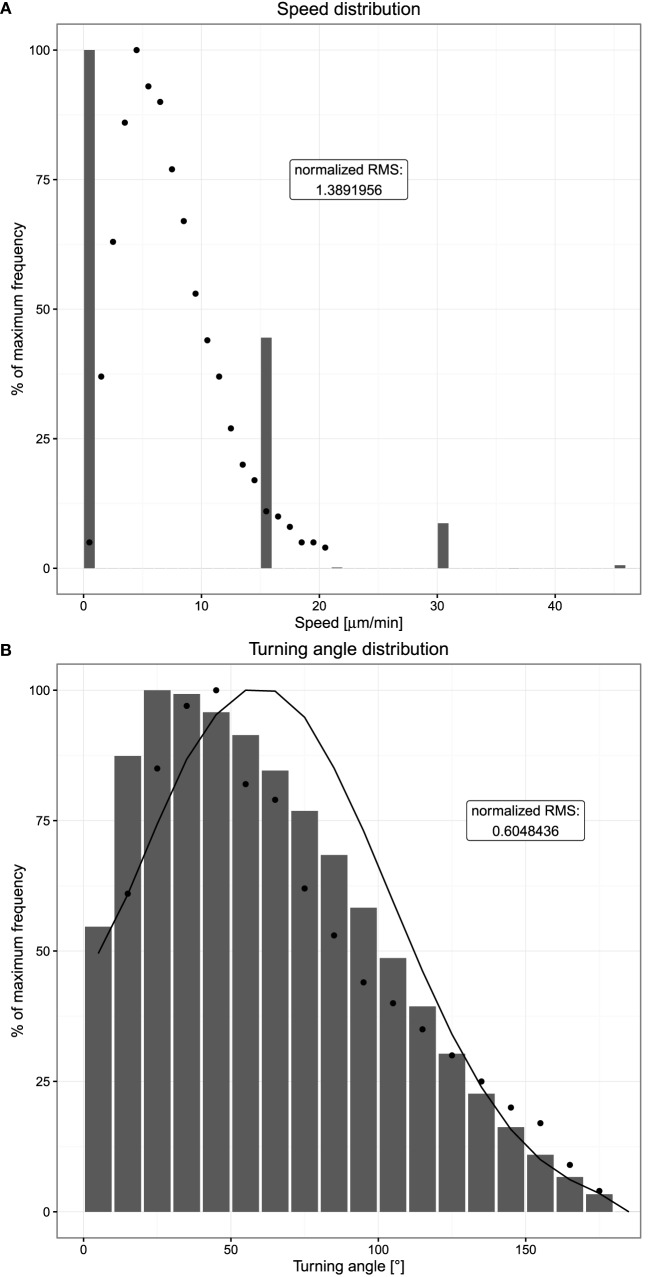
**Speed and turning angle distributions of B cells**. Dots indicate experimental measurements, and bars show the mean of 20 simulations. **(A)** Distribution of observed speeds expressed as percent of maximum frequency. **(B)** Distribution of observed turning angles expressed as percent of maximum frequency. The distribution indicated by the solid line shows the mean of 20 simulations without chemotaxis. Cells are tracked every 20 s both *in silico* and in experiment.

*In silico*, the speed distribution based on time intervals was mimicked. This induces basically a reflection of the lattice architecture, populating speeds that correspond to the distances of next and next-to-next neighbors on the lattice divided by the chosen time interval (Figure 3A). When increasing the time interval to the range of the persistence time, the distribution spreads *in silico* and reaches a smooth distribution at intervals of 10 min (data not shown). In this range, the observed mean speed gets lower because more and more performed movements are neglected by the large time interval, which imposes that only the start and end positions are compared.

The smooth distribution at larger time intervals exhibits the asymmetry with more low speed events and less high speed events than found in experiment (data not shown). *In silico*, the asymmetry reveals repolarization events that limit the distance reached within a single time interval. The longer the time interval, the more the repolarization events and the lower the derived speed.

In comparison to the experimental speed distribution, a large peak at zero speeds is observed *in silico*. This reflects a probability of not moving on the lattice. With a speed of 7.5 µm/min and a time interval of 20 s, the cell should move 2.5 µm/time interval. With a lattice constant of 5 µm, this would mean that a cell moves in about 50% of the cases. This is approximately found in the *in silico* speed distribution.

In real experiments, cells interacting with other cells would not sit at fixed positions but wiggle around, always leading to finite speeds larger than zero. Zero speed counts are hardly found in experimental data. In contrast, *in silico* B cells, once in contact to either FDCs or Tfh stop moving. B cells also do not move during M phase of the cell cycle. We therefore asked how large the impact of these frozen cells is onto the speed distribution. Ignoring cells in contact with other cells in the movement analysis reduces the zero speed count by less than 5%. We conclude that the speed distributions are not dominated by cell–cell interactions *in silico* or wiggling cells *in vivo*.

### 3.3 Turning Angle Distribution

The turning angle distribution measures the changes in directionality of cells. Cells round up in experiment and then choose a new direction. Considering cells to walk on a dendritic cell network, which can be approximated by a hexagonal mesh, one would expect a turning angle of 60° to be the most frequent. The experimental measurement shows that the most frequent angle is even lower. Again an asymmetric distribution is found.

*In silico*, the turning angle of a cell is assumed to be sampled from a Gaussian centered at 60°, thus from a symmetric distribution. Despite this, the turning angle distribution measured *in silico* is asymmetric (Figure 3B). This reflects the impact of chemotaxis: without any chemotactic gradient, B cells show a random walk behavior where a new polarity is simply a random variable. If a chemotactic gradient is present and within the sensitive region, a chemotactic component is added to the random polarity vector, introducing a bias toward smaller turning angles. Hence, the average observed turning angle is smaller if chemotaxis is present; indeed, switching off chemotaxis leads to a symmetric distribution (Figure 3B).

Note that the discretization by the lattice does not impose all cells to turn with angles of 90° because the repolarization after each persistence time induces a new polarization vector, which is not restricted to the directions of the lattice. Thus, the *in silico* cell might move diagonally or at any other angle by choosing a right or left turn of 90° with corresponding probabilities. The resulting turning angle distribution *in silico* is as smooth as that in *in vivo*. However, a relevant difference between the experimental measurements and the simulations is the observed behavior at very small turning angles. While in the *in vivo* system, the occurrence of very small turning angles close to 0°can be expected to be a rather rare event, it is observed frequently in simulations due to the lattice structure. Hence, we consider an accumulation of 0°turning angles to be an artifact of the space discretization and considered turning angles of more than 10° only in order to allow comparison of simulation and experimental data.

We have previously published that the turning angle distribution, in contrast to the speed distribution, is widely invariant against choices of the time interval of cell measurements (18). Therefore, this distribution can be considered as a real property of the cells in their microenvironment.

### 3.4 Transzone Migration

**Figure 4:**
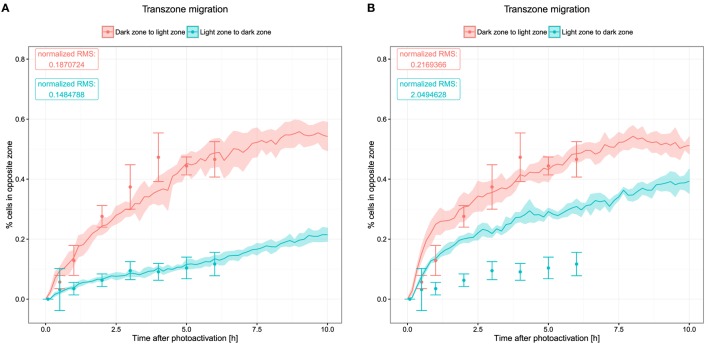
**Transzone migration as measured by tracking photoactivated cells *in silico***. Photoactivation is performed in a defined region in either of the two zones and subsequent migration of the photoactivated cells is tracked. Displayed data show the fraction of photoactivated cells that appear in the opposite zone. Dots and error bars indicate means and SD from experimental measurements (8), lines and shaded area show mean and SD from 20 simulations. **(A)** Transzone migration with reference chemotaxis sensitivity parameters (see Figure 1); **(B)** transzone migration in simulations with alternative parameters for chemotaxis (see Figures 5C,D).

Transzone migration was measured by tracking GC B cells photoactivated in either zone and analyzing how many tracked cells are found in the opposite zone over the time period of 6 h (8). This data set shows a strong asymmetry between both directions of transzone migration, with LZ to DZ transitions being less frequent (Figure 4). While this could be interpreted as a sign against recycling, this asymmetry was matched by the LEDA model assuming a recycling probability of 80% or more (20). The reason for this robustly observed asymmetry is the selection process of B cells in the LZ. Either in the DZ or the LZ, a fixed number of cells are photoactivated initially. While all of the photoactivated GC DZ cells at some point will migrate to the LZ for selection, only the subset of selected GC LZ cells migrates to the DZ for another round of division. Furthermore, apoptotic centrocytes remain visible for some time before they are phagocytosed. In this stage, they do not upregulate CXCR4 anymore and, hence, cannot migrate to the DZ, thus contributing further to a lower LZ to DZ migration rate.

The new model, which includes a continuous influx of B cells, thus, embedding B cell clones with different stages of history as well as the DND mechanism that might change the proportion of cells passing between both zones, is still in agreement with these data (Figure 4A). The asymmetry of transzone migration events can be considered as a robust property of GC simulations.

**Figure 5:**
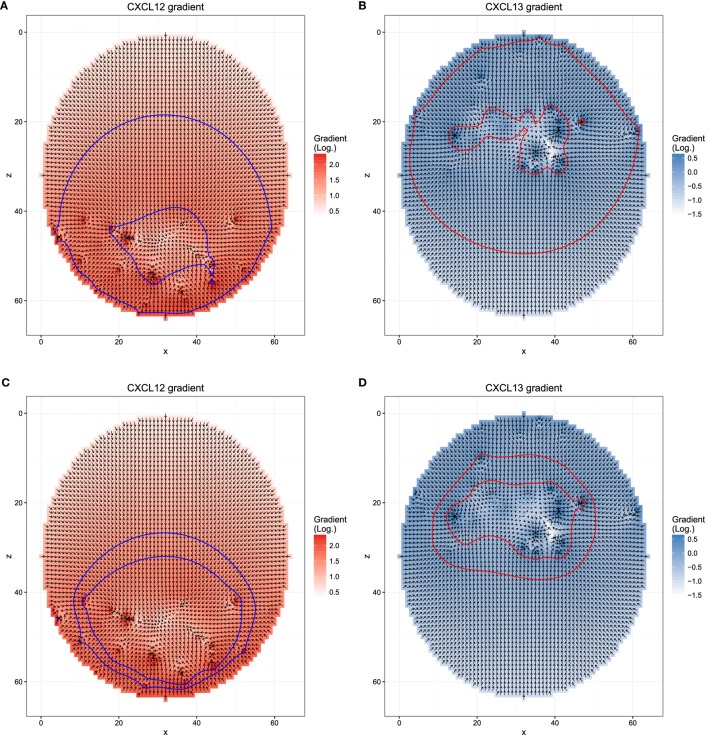
**Different chemokine distributions that lead to reduced asymmetry in the transzone migration *in silico***. Figures show the chemokine gradient in a two-dimensional cutsection through a germinal center. Gradient directions are indicated by the arrows in the vector field plots, its magnitude is shown by the color. Lines indicate the positions at which desensitization (inner border) and resensitization (outer border) occur. **(A,B)** Chemotaxis with resensitizations at lower concentrations compared to the reference simulation; **(C,D)** chemotaxis with desensitization at the concentration at the zone boundary and resensitization at a 30% lower concentration.

Next, we asked whether the asymmetry of transzone migration is also robust against modifications in the transient chemotaxis model. In order to test this, resensitization was assumed at lower concentrations (Figures 5A,B). Alternatively, desensitization was set to the value at the zonal boundary and resensitization at 30% lower concentrations (Figures 5C,D). The asymmetry is lost in both cases (Figure 4B) because cells from either zone transmigrate to the other zone, not because of a changed phenotype but just by random walk. This process is dominant and hides the asymmetry found in the few cells that changed the phenotype from centroblast to centrocyte or vice versa and subsequently transmigrated to the other zone.

The transzone migration behavior is sensitive to the choice of the lattice constant: choosing a lower resolution with a lattice constant of 10 µm tends to inhibit the movement of the cells in general leading to a more densely packed simulation grid. Cells that occupy a node on the grid do not block movement completely, since two cells can exchange their position under certain circumstances. Specifically, if two cells attempt to move in opposite directions, i.e., the scalar product of their polarity vectors is negative, the cells are exchanged with a probability of 0.5. However, since this type of movement is far more restricted than movement to free grid positions, a crowded grid significantly inhibits cell movement *in silico*. In particular, this is reflected in the transzone migration behavior, where a larger lattice constant reduces the asymmetry of transzone migration events.

We conclude that the asymmetry of transzone migration events is robust against details of selection mechanisms and cell immigration, but not against the particular chemokine profile and the dynamics of cell sensitivity for chemotaxis.

### 3.5 DZ to LZ Ratio

The DZ to LZ ratio is a stable property of GCs, even between different species (14). It is also stable over time; thus, this ratio appears in a flow equilibrium of division, selection, and transzone migration.

Using the DEC205 receptor expressed on B cells, Victora and colleagues had the intriguing idea to provide the antigen (ovalbumin) with the right specificity via this receptor (8). Ovalbumin was bound to the ligand of DEC205 and injected. The subset of DEC205-positive cells now had a competitive advantage of being selected, because these cells got the antigen for free, not needing to collect it from the FDC network. Indeed, peptide MHC presentation of these cells was found 5-fold higher than in B cells collecting antigen from FDCs.

**Figure 6:**
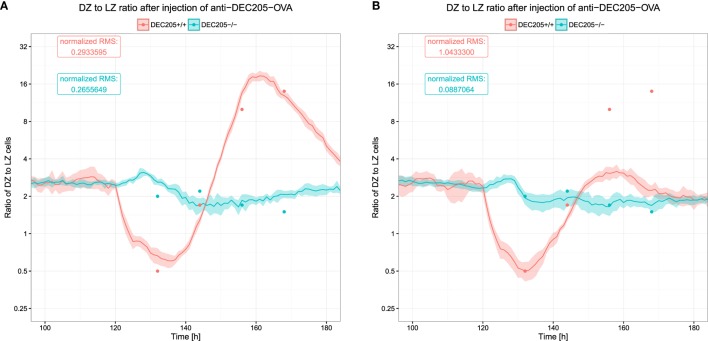
**Ratio of B cells in the dark zone to those in the light zone after injection of *α*DEC205-OVA**. Dots indicate experimental measurements (8), lines and shaded area show mean and SD from 20 simulations. The two colors show the reaction of DEC205^+/+^ and DEC205^−/−^ cells. DEC205^−/−^ are not subjected to stimulation by *α*DEC205-OVA and can, thus, be expected to represent the behavior in the absence of strong antigenic stimulation. **(A)** DZ to LZ ratio in reference simulations with DND; **(B)** DZ to LZ ratio in simulations without DND.

The DZ to LZ ratio was used to measure the reaction of the DEC205-positive GC B cells and showed large fluctuations (see Figure 6). *In silico*, these fluctuations were only recapitulated when B cells would reside for longer times in the phase of interaction with Tfh and when the number of divisions attributed to the DEC205-positive subset of B cells was multiplied by 2.3, leading to the prediction of a dynamically regulated peptide MHC density-dependent number of divisions that should induce division numbers between 1 and 5 or 6 (20).

It was shown that such a DND effect really exists, depends on the amount of presented antigen (21), and has evolutionary advantages (27). Therefore, it is relevant to quantitatively determine the parameters controlling the relation of antigen presentation on B cells and the number of divisions induced by help from Tfh cells. In this model, the number of divisions is derived from the amount of collected or offered antigen by a Hill function (see Section 2). We identified the quantitatively correct parameters (Table 1) of the Hill function that reproduce the DZ to LZ dynamics upon anti-DEC205-ovalbumin treatment (Figure 6A). In contrast, switching off DND produces markedly different results that conflict with experimental measurements (Figure 6B). The parameters identified in this way are *P_min_* = 1, *P_max_* = 6, *n_P_* = 1.3, and *K_P_* = 11.6194. Note that the minimum and maximum values of DND, *P*_min_ and *P*_max_, respectively, are determined by the experimental results in Ref. (21) and were not varied. The antigen amount corresponding to the half value of DND was derived from the side condition that the mean number of divisions should match the mean number of divisions found in real GCs, which is in the range of two divisions (21). The *K_P_* value that meets this condition depends on the Hill coefficient *n_P_*, which is the only free parameter remaining. As only one parameter was free in this fitting procedure, it can be considered as identifiable by the data set of DZ to LZ ratios.

**Table 1:** **Parameters and values for the affinity-dependent number of B cell divisions (DND)**.

Parameter	Description	Value
*P*_min_	Minimal number of divisions	1
*P*_max_	Maximal number of divisions	6
*n_P_*	Hill coefficient	1.3
*K_P_*	Number of antigen collection events required for half of maximum divisions	11.619

## 4 Discussion

The analysis of the available two-photon measurements of GC B cell migration is compatible with the LEDA model extended by antibody feedback and continuous influx of founder B cells. The described GC model has been significantly extended since earlier publications according to novel experimental results that warranted the inclusion of several new mechanisms. While the newly introduced mechanisms are well supported by experiments, this work presents the first systematic comparison of this fundamentally changed model to important experimental data sets from live imaging studies.

The DZ to LZ ratio in response to antigen delivered to a subset of GC B cells was able to determine the strength of competitive advantages in the interaction between Tfh and B cells. For the first time, we quantitatively characterized this mechanism. We found a steady increase in the number of divisions with the density of presented peptide MHC, which was best described by an almost linear Hill function with Hill coefficient *n_P_* = 1.3. While the DND effect is essential to understand the dynamics of affinity maturation and take over by high-affinity clones, DND turns out to reflect a rather smooth dependence on the quality of the B cell and its efficiency in collecting, processing, and presenting peptide on MHC.

Recent measurements of affinity maturation suggested that selection is possibly less stringent than believed so far (34). The present finding that low- and high-affinity B cells would not induce a big difference in the number of induced divisions further reduces the stringency of selection and fosters the view that GC evolution is influenced by random processes. At the same time, this would infer a larger diversity of GC outcomes in terms of dominance of GCs by single clones as demonstrated by recent brainbow experiments (25).

The second important result of the analysis is the observed robustness of the asymmetry of transzone migration of LZ and DZ cells to the respective opposite zone. This asymmetry emerges not only for different degrees of the recycling probability (20) but is also robust against changes in the DND effect. It is equally recovered in settings with constant numbers of divisions induced by Tfh or with even more stringent DND effects than derived from the DZ to LZ data. In contrast, this asymmetry relies on a strict separation of DZ and LZ cells (16). If the zones are too leaky, the rare transzone migration events are fully dominated by the random transzone migration events in both directions. Therefore, the transzone migration asymmetry can be considered as a signature for the chemotaxis profiles keeping B cells in their respective zone.

In this model, Tfh were considered motile and sensitive to CXCL13, thus, predominantly residing in the LZ. Tfh influx or egress from the GC was observed (22), but was not considered here. In particular, CXCL13 production by Tfh was not considered. This assumption might be a limitation of the model; CXCL13 production has been clearly shown for human Tfh (35–37). While no CXCL13 expression was found in earlier studies on murine Tfh (38), recent data found a correlation between CXCL13 in blood and the fraction of Tfh among CD4^+^ cells (35). We do not expect major changes in the CXCL13 distribution by Tfh-produced CXCL13 because Tfh would produce CXCL13 in the LZ where the FDCs, the included sources of CXCL13, are residing anyway. In a possible extension of the model including more detailed dynamics of Tfh, this expectation could be tested.

The photoactivation experiment was initiated at day 6 and the anti-DEC205-OVA experiment at day 5 post GC onset (8). One might ask an important question, whether the results would rely on the particular time point of performing these experiments. This becomes even unlikely in view of these time points being around the peak population of the GC B cells. Furthermore, these time points correlate with a highly dynamic phase of affinity maturation, which would also impact on the number of divisions attributed to B cells and on the amount of competition for Tfh help. We asked whether the simulations would suggest a dependence of these results on the time point of experimentation and repeated the *in silico* experiments from days 3 to 11 post GC onset. Intriguingly, both, asymmetry of transzone migration and DZ to LZ dynamics in response to anti-DEC205-OVA remained the same for all later time points (data not shown). This is surprising, because the total number of B cells at day 11 post GC onset dropped to 50% and affinity maturation was completed; thus, almost all GC B cells showed a high affinity at this time. However, the DZ to LZ ratio in response to anti-DEC205-OVA injection at days 3 or 4 post GC onset did not exhibit the accumulation of GC B cells in the LZ, as observed for later time points. In this early phase of the GC reaction, the GC is still dominated by B cells in the expansion phase, thus, outweighing the subtle effects in the LZ from the artificially provided antigen. We conclude that spatial parameters can be robustly assessed by multiphoton imaging of GC reactions, as long as the time point of measurement is well separated from the expansion phase. If one is not particularly interested in the dynamics of the expansion phase itself, the simulations suggest such experiments at day 8 post-immunization or later.

We are convinced that there is a large mutual benefit of joining two-photon experiments with mathematical models. The simulations provide insights into the motility of the observed cells (or other objects). In view of interaction patterns and complex dynamics, the motility measurements can be set into a larger context by the simulations. In other words, the physiological impact of a particular motility pattern can be unraveled much easier by using mathematical models as an interpretation tool.

## Author Contributions

SB and MM-H designed the study, programed the code, analyzed the data, drew the conclusions, and wrote the article.

## Conflict of Interest Statement

The authors declare that the research was conducted in the absence of any commercial or financial relationships that could be construed as a potential conflict of interest.
